# *Bartonella hanselae* retinitis patient evaluated with multimodal retinal exams

**DOI:** 10.1186/s40942-020-00257-6

**Published:** 2020-11-13

**Authors:** Paulo Alberto Cervi Rosa, Luis Filipe Nakayama, Vinicius Campos Bergamo, Dante Akira Kondo Kuroiwa, Nilva Simeren Bueno de Moraes

**Affiliations:** Physician Department of Ophthalmology, Universidade Federal de São Paulo - EPM, Botucatu St., 821, Vila Clementino, São Paulo, 04023-062 Brazil

**Keywords:** Bartonella, Multimodal imaging, Retinitis

## Abstract

**Background:**

Cat scratch disease is a systemic infectious illness caused by the bacterium *Bartonella henselae*. The most common ophthalmological involvement due to infection by *Bartonella* is Parinaud oculoglandular syndrome, whereas the most common posterior segment findings are neuroretinitis and subsequent late macular star. Moreover, other findings, such as retinal or subretinal lesions, intermediate uveitis and angiomatous lesions, may be present.

**Case presentation:**

A 37-year-old female patient with retinal findings and serological confirmation of *Bartonella* infection was evaluated via multimodal retinal exams. The patient received treatment with doxycycline 100 mg twice daily for 2 weeks. One month after treatment, complete improvement of her visual scotoma symptoms was confirmed. A swept-source optical coherence tomography exam also showed decreases in the size and intraretinal extension of the lesion. Improvement of light perception at the affected area was confirmed by microperimetry.

**Conclusions:**

*Bartonella henselae* infection, particularly retinitis, can present a variable spectrum of clinical and ophthalmological findings. Multimodal retinal exams can clearly identify lesion characteristics, thus providing important information for diagnosis and the evaluation of lesion improvement after antibiotic treatment.

## Background

Cat scratch disease is a systemic infectious illness caused by the bacterium *Bartonella henselae*, a gram-negative bacillus responsible for a disease with a variable clinical spectrum [1–3].

The most common ophthalmological manifestation of *Bartonella* is Parinaud oculoglandular syndrome, first described in 1889, which presents as ulcerative chronic conjunctivitis and lymphadenopathy [1–3]. The most common posterior segment findings are a swollen optic disc, neuroretinitis and subsequent late macular star [1, 4], but other findings may be present, such as retinal or subretinal lesions, intermediate uveitis and angiomatous lesions [3].

This case report describes a female patient with retinal findings and serologically confirmed *Bartonella* infection who was evaluated with multimodal retinal exams, including retinography, swept-source optical coherence tomography (SS-OCT), fundus autofluorescence (FAF), fluorescein angiography (FA), angio-OCT, and microperimetry, before and after antimicrobial treatment.

## Case report

A 37-year-old female presented at the emergency sector with a chief complaint of paracentral visual scotoma in her left eye for 3 weeks. She referred that the scotoma occurred suddenly and that this symptom was steady and painless. A couple days prior to the onset of the visual complaint, she had a mild headache and unverified fever.

**Fig. 1:**
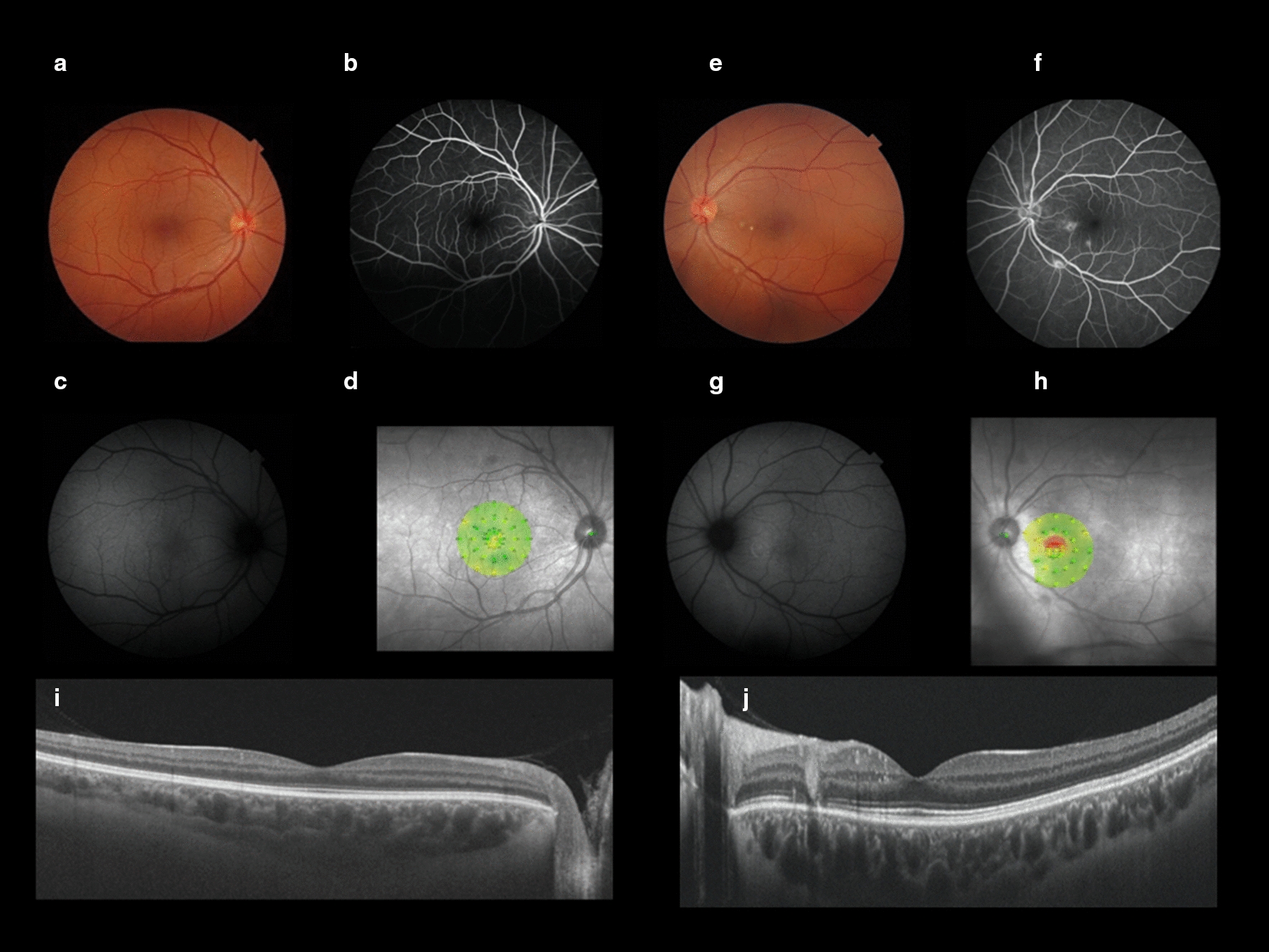
Pre-treatment multimodal retinal imaging: **a** Retinography of the right eye **b** Fundus angiogram of the right eye—mid arteriovenous phase, showing staining, but not leakage, pattern of the lesions. **c** Fundus autofluorescence of the right eye. **d** Microperimetry of the right eye. **e** Retinography of the left eye—two white lesions in papillomacular bundle and one white lesion in inferotemporal arcade. **f** Fundus Angiogram of the left eye—staining, but not leakage, pattern of the lesions. **g** Fundus autofluorescence of the left eye—Hyper Autofluorescence at retinal lesions. **h** Microperimetry of the left eye. **i** Swept Source OCT of the right eye. **j** Swept Source OCT of the left eye—well delimited intra retinal lesions at deep and intermediate layers

Upon ophthalmological examination, she presented with best corrected visual acuity of 20/20 in both eyes and intraocular pressure of 14 mmHg (OD) and 15 mmHg (OS). Anterior biomicroscopy revealed a clear cornea, clear conjunctiva, deep anterior chamber, no anterior chamber reaction and no vitreous cells in both eyes. The retinal fundoscopic exam indicated one small (0.1 optic disc diameter) white round lesion in the superior nasal vessels area of the right eye and two small parafoveal round nasal yellowish lesions and one small white lesion at the inferior temporal vessels area of the left eye (Fig. 1). No restriction of ocular motility was observed. Pupillary light reflex examination showed normal direct and consensual pupillary reflexes and no relative afferent pupillary defect in both eyes.

As retinal ancillary exams, color fundus retinography, SS-OCT (Swept source optical coherence tomography), FAF (fundus autofluorescence), FA (fluorescein angiography), angio-OCT and microperimetry were performed. General ancillary exams included general tests such as systemic serum laboratory workup, thorax radiography and infectious serologies.

SS-OCT showed preserved retinal layers in macular topography and well-limited intraretinal lesions extending from superficial to deep retinal layers (Fig. 1). Epidemiology included contact with newborn cats, leading us to hypothesize Bartonella henselae infection. FAF showed hyperautofluorescence in areas corresponding to the retinal lesion margins without any other significant findings (Fig. 1). FA showed a late fluorescence enhancement at areas corresponding to the retinal lesions that increased during the course of the exam but with limited margins, consistent with the staining pattern (Fig. 1). Similar findings in OCT and FA were previously reported in medical literature as being caused by Bartonella infection [3, 5]. Objective evaluation by microperimetry of the macular scotoma cited as the chief complaint of the patient showed a decrease in light perception at areas corresponding to the retinal lesions (Fig. 1).

The first diagnostic hypothesis considered was chorioretinopathy of both non-infectious causes, such as punctate inner choroidopathy (PIC), multiple evanescent white dot syndrome (MEWDS), and sarcoidosis, and infectious causes, such as tuberculosis, syphilis, toxoplasmosis or bartonellosis.

Her laboratory workup included non-reactive serologies for syphilis, HIV and toxoplasmosis, tuberculin skin-test of zero, non-reactive anti-nuclear factor, reactive c-protein of 7 (reference value of 1) and erythrocyte sedimentation rate of 44 (reference value of 20). Her complete blood count exam reported normal red blood cell and platelet values and mild leukocytosis due to mild neutrophilia (15,500 leukocytes with 55.5% neutrophils, 37.0% lymphocytes, 4.9% monocytes, 1.7% eosinophils, 0.9% basophils) but without a left shift. Thorax radiography did not show significant findings. *Bartonella henselae* serology was positive, with an IgG of 1/128, and treatment with 100 mg doxycycline every 12 h for 2 weeks was administered.

**Fig. 2:**
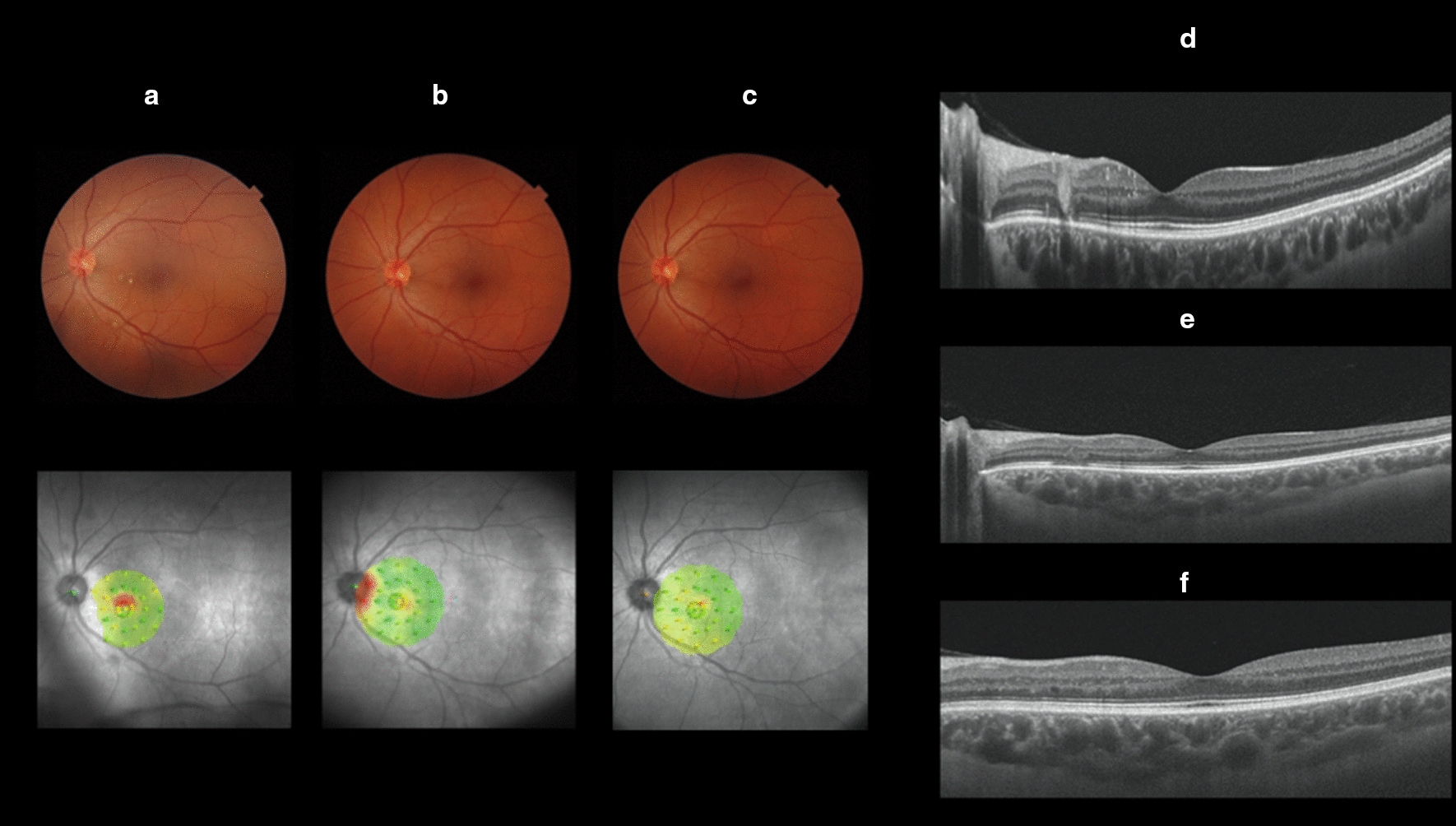
Comparison of pre and post-treatment multimodal retinal imaging of the left eye. **a** Retinography and microperimetry (pre-treatment), **b** Retinography and microperimetry (1 month post-treatment). **c** Retinography and microperimetry (18 months post-treatment). **d** Swept Source OCT (pre-treatment). **e** Swept Source OCT (1 month post-treatment)—external retinal layers disorganization and interruption. **f** Swept Source OCT (18 months post-treatment)—mild disorganization of intermediate retinal layers at the site of the previous lesion surrounded by a halo

One month after treatment, she reported an improvement of visual scotoma symptoms. At her retinal fundus examination, it was possible to locate only one of the previous lesions (paracentral of her left eye), and the presentation of this lesion was more subtle than at the first evaluation. SS-OCT also showed decreases in lesion size and intraretinal extension. Microperimetry showed an improvement in light perception in the affected area (Fig. 2).

Eighteen months after the initial presentation, the patient underwent another evaluation. She had no visual complaints and her ophthalmologic exam was normal. There were no more signs of retinal lesions at the fundoscopy. Microperimetry showed a greater improvement in light perception at the previous affected area and SS-OCT showed only a mild disorganization in intermediate retinal layers at the site of the previous lesion surrounded by a halo (Fig. 2).

## Discussion

The classic presentation of *Bartonella henselae* neuroretinitis is a swollen optic disc and macular edema as described by Sweeney and Drance in 1970, but this disease may present a variable spectrum of clinical and ophthalmological findings [1, 4]. This case report is of a female patient with bilateral intraretinal lesions; normal blood and general exams; negative serology for human immunodeficiency virus, syphilis and toxoplasmosis; positive epidemiology for contact with cats; and positive serology for *Bartonella henselae*.

Multimodal retinal exams helped to clearly identify the lesions’ characteristics as well as their improvement after antibiotic treatment. SS-OCT, angio-OCT and en-face evaluation facilitated the determination of the exact lesion topography and the exclusion of other macular involvement. FAF and FA enabled the evaluation of the lesions’ characteristics and the exclusion of other conditions, such as neuritis and vasculitis. Microperimetry examination objectively detected visual scotoma and subsequent improvement in both eyes. All exams showed improvement at one month follow-up after antimicrobial therapy, with a decrease in lesion size and improvement of visual scotoma based on microperimetry. Eighteen months after the initial presentation, multimodal retinal evaluation showed an even greater improvement of the retinal involvement and light perception at the affected area of the previous lesions.

*Bartonella henselae* infection, particularly retinitis, can present as a variable spectrum of clinical and ophthalmological findings. It is important, as soon as this etiology is suspected, to actively investigate and question the epidemiologic exposure, since it is an information that is usually neglected by patients during interrogation and that helps on the formulation and corroboration of the diagnostic hypothesis, especially if it is a positive finding [3]. Multimodal retinal exams aided the clear identification of lesion characteristics in a patient who sought medical care due to visual symptoms compatible with scotoma, thus providing important information for the diagnosis formulation and for the evaluation of lesion improvement after antibiotic treatment, such as the precise localization of the lesions and its characteristics (dimension and intraretinal extension). After treatment, a residual scotoma in the region of the lesion with a surrounding halo was observed. We hypothesise that it may be a characteristic of lesions with a worse prognosis and that lead to visual sequelae despite a reduction in size due to treatment and clinical evolution.

To the best of our knowledge, this is the first report of the evaluation of *Bartonella henselae* retinitis with multimodal retinal exams before and after treatment, which led to an objective improvement in symptoms.

In summary, we report the utility of multimodal retinal exams in the diagnosis, follow-up, and improved characterization of a retinal infectious disease, particularly for an atypical case in which other possible diagnoses have to be considered.

## Data Availability

Not applicable.
